# Identification of Small RNAs During High Light Acclimation in *Arabidopsis thaliana*

**DOI:** 10.3389/fpls.2021.656657

**Published:** 2021-06-15

**Authors:** Bhavika Tiwari, Kristin Habermann, M. Asif Arif, Oguz Top, Wolfgang Frank

**Affiliations:** Plant Molecular Cell Biology, Department of Biology I, LMU Biocenter, Ludwig-Maximilians-University Munich, Planegg-Martinsried, Germany

**Keywords:** *Arabidopsis thaliana* (Arabidopsis), high light acclimation, small non-coding RNA, gene regulation, RNA sequencing

## Abstract

The biological significance of non-coding RNAs (ncRNAs) has been firmly established to be important for the regulation of genes involved in stress acclimation. Light plays an important role for the growth of plants providing the energy for photosynthesis; however, excessive light conditions can also cause substantial defects. Small RNAs (sRNAs) are a class of non-coding RNAs that regulate transcript levels of protein-coding genes and mediate epigenetic silencing. Next generation sequencing facilitates the identification of small non-coding RNA classes such as miRNAs (microRNAs) and small-interfering RNAs (siRNAs), and long non-coding RNAs (lncRNAs), but changes in the ncRNA transcriptome in response to high light are poorly understood. We subjected *Arabidopsis* plants to high light conditions and performed a temporal in-depth study of the transcriptome data after 3 h, 6 h, and 2 days of high light treatment. We identified a large number of high light responsive miRNAs and sRNAs derived from NAT gene pairs, lncRNAs and *TAS* transcripts. We performed target predictions for differentially expressed miRNAs and correlated their expression levels through mRNA sequencing data. GO analysis of the targets revealed an overrepresentation of genes involved in transcriptional regulation. In *A. thaliana*, sRNA-mediated regulation of gene expression in response to high light treatment is mainly carried out by miRNAs and sRNAs derived from NAT gene pairs, and from lncRNAs. This study provides a deeper understanding of sRNA-dependent regulatory networks in high light acclimation.

## Introduction

Acclimation to changing abiotic and climatic conditions is a prerequisite for plants to survive. High light stress is probably the most frequently experienced stress by plants and efficient light utilization requires proper acclimation to light-limiting and light-excess conditions. To counter the effects of high light, plants respond systemically by adjusting leaf orientation, depositing salt crystals on the leaf surface or developing air-filled hairs (Ruban, 2009).

Under excess light, photoinhibition provokes the production of reactive oxygen species (ROS) which leads to inactivation of the PSII reaction center by photodamage (Ohnishi et al., 2005; Murata et al., 2007). In the last few years, studies revealed that plants have developed mechanisms to cope with photodamage such as thermal dissipation of excess energy, xanthophyll cycle, cyclic electron flow and photorespiratory pathways (Demmig et al., 1987; Park et al., 1996; Niyogi et al., 1998; Cornic et al., 2000; Maxwell and Johnson, 2000; Wingler et al., 2000; Clarke and Johnson, 2001; Munekage et al., 2004; Miyake et al., 2005; Jahns and Holzwarth, 2012).

When plants are exposed to high light, chloroplasts transmit retrograde signals to the nucleus (Nott et al., 2006) in order to downregulate the expression of photosynthesis associated genes and to induce defense related genes to prevent oxidative damage (Apel and Hirt, 2004; Van Breusegem et al., 2008). In a previous study, the phytohormone abscisic acid (ABA) was found to be essential to coordinate the expression of high light responsive genes in coordination with retrograde signaling mechanisms (Valdivieso et al., 2009). During high light stress, singlet oxygen (^1^O_2_) which is generated due to imbalanced redox potential induced the expression of β-cyclocitral, about 10 glutathione S-transferase and 12 UDP-glycosyltransferase genes. These genes are known to be involved in detoxification of endogenous compounds such as lipid peroxides and to confer stress tolerance to ^1^O_2_ (Ledford et al., 2007).

Due to excess light, the electron transport chain is over reduced and PSII can be affected by photoinhibition. This imbalance of redox potential leads to the production of high amounts of ^1^O_2_ in PSII that can cause the formation of irreversible reactive oxygen species (ROS), peroxides, and radical induced damages even though high levels of ROS have also been shown to act in signaling pathways in response to high light (Karpinski et al., 2003). Plants have evolved mechanisms to protect themselves from elevated ROS levels by ROS scavenging proteins such as ascorbate peroxidases (APX), superoxide dismutase (SOD), glutathione peroxidases (GPX), catalases (CAT), and peroxiredoxins (PRX). The reduced levels of fluorescence quenching (NPQ) was found to be a potential initiator of ROS production such as H_2_O_2_ and subsequent increase in ROS levels were found to be the regulators of *APX1* and *APX2* encoding ascorbate peroxidases (Szechynska-Hebda et al., 2010). The non-enzymatic antioxidants include ascorbate and glutathione, flavones, carotenoids, tocopherols and anthocyanins (Birben et al., 2012). The constant process of ROS production and scavenging occurs in all cellular compartments and hence is tightly controlled by a ROS associated gene network (Mittler et al., 2004).

In addition to transcriptional changes of protein-coding genes light stress also causes changes in the expression of non-coding transcripts (Wang H. et al., 2014). Non-coding RNAs (ncRNAs) are divided into two groups based on their size. ncRNAs shorter than 200 nt are considered as small ncRNAs whereas longer transcripts are referred to as long ncRNAs. Among the small ncRNAs miRNAs with a size of approximately 21 nt are prominent regulators of gene expression. miRNAs are transcribed as primary miRNAs from *MIR* genes by RNA polymerase II. The pri-miRNA folds back into a stem loop structure which is further processed into a pre-miRNA by DICER-LIKE1 (DCL1), HYPONASTIC LEAVES1 (HYL1), and SERRATE (SE) that is further processed to release the mature miRNA:miRNA^∗^ duplex (Voinnet, 2009). The duplex becomes 3′ methylated by HUA ENHANCER1 (HEN1) protecting the miRNA from degradation (Yu et al., 2005; Gao et al., 2021). The mature miRNA strand binds to ARGONAUTE1 (AGO1) and is loaded into the RNA-induced silencing complex (RISC) guiding the complex to fully or partially reverse complementary target transcripts causing target cleavage or translational inhibition (Voinnet, 2009).

Studies in different plant species have been conducted to identify differentially expressed miRNAs in response to high light, UV-A and UV-B (Zhou et al., 2016; Millar, 2020). miR156/157, miR167, miR170/171, and miR159/319 are known to be red light and UV-B responsive in *Arabidopsis* (Zhou et al., 2007; Tsai et al., 2014; Zhenfei et al., 2018), *Oryza sativa* (Sun et al., 2015), *Glycine max* (Li et al., 2015) and *Triticum aestivum* (Wang et al., 2013). The expression levels of miR165/166, miR396, miR408, and miR169 were UV-B, white and red light regulated in *A. thaliana*, *O. sativa* and *G. max* (Casadevall et al., 2013). miR398, miR172, miR160, miR169, miR164, miR395, miR399, miR168, miR393, miR858, miR163, miR390, and miR397 were responsive to white and far red light, UV-A, UV-B and differentially expressed in *Arabidopsis phyB* (*phytochrome B*) and *pif4* (*phytochrome interacting factor 4*) mutants (Chung et al., 2016; Sharma et al., 2016; Lin et al., 2017; Zhenfei et al., 2018). In addition, miR396 was found to be upregulated in response to UV-B light mediating the downregulation of its targets encoding *GROWTH REGULATING FACTOR1 (GRF1)*, *GRF2*, and *GRF3* that led to an inhibition of cell proliferation in leaves (Casadevall et al., 2013). miR163 was also found to be highly induced by red light in *Arabidopsis* targeting *PXMT1* encoding a 1,7-paraxanthine methyltransferase involved in methylation of phytohormones (Chung et al., 2016). In the early stages of development, this miRNA and its target were also found to regulate germination. Upregulation of miR156 was found to be important for increasing anthocyanin levels in *Arabidopsis.* miR156 targets *SPL* transcripts which are known to repress the anthocyanin biosynthesis pathway (Gou et al., 2011; Cui et al., 2014). In addition to miR156, miR858 is also considered a positive regulator of anthocyanin biosynthesis as it targets *MYBL2* coding for a repressor of the phenylpropanoid pathway (Sharma et al., 2016; Wang et al., 2016b). ELONGATED HYPOCOTYL 5 (HY5) was also shown to be a positive regulator of the anthocyanin pathway as it downregulates MYBL2 (MYB-LIKE 2) (Nguyen et al., 2015).

lncRNAs can be transcribed from opposite DNA strands generating overlapping sense and antisense transcripts referred to as natural antisense transcripts (NAT). When lncRNAs do not overlap with any protein coding gene, but are present between two genes or in the intronic region, they are referred as long intergenic or intronic non-coding RNAs (lincRNAs) (Ma et al., 2013). Recent studies have found lncRNAs involved in light regulated processes, *HIDDEN TREASURE 1* (*HID1*) was found to act through PIF3 which is a key repressor of photomorphogenesis (Wang Y. et al., 2014) and *CDF5 LONG NON-CODING RNA (FLORE)*, a NAT of *CDF5* repressed *CDF5* itself and promoted transcription of *FLOWERING LOCUS T* (*FT)* which induced flowering (Henriques et al., 2017). The RNA polymerase II derived NATs are able to produce siRNAs from overlapping regions referred to as nat-siRNAs. Depending on the genomic locations of the two overlapping transcripts, the NATs are classified as *cis*-NATs when the transcripts are encoded by complementary DNA strands at the same genomic region and referred to as *trans*-NATs when the transcripts are produced from two different regions in the genome (Wight and Werner, 2013). The first identified *cis*-nat-siRNA producing loci have an important role in response to high salinity stress. The constitutively expressed transcript *delta-pyrroline-5-carboxylate dehydrogenase* (*P5CDH*) and the salt induced transcript *Similar to Radicle Induced Cell Death One 5* (*SRO5*) produce 24 nt *cis*-nat-siRNA from a dsRNA formed by both transcripts. The AGO protein facilitates the *cis*-nat-siRNAs to cleave the *P5CDH* transcript resulting a decreased proline degradation and improved salinity tolerance (Borsani et al., 2005). *trans*-nat-siRNAs are produced in a similar manner, but transcript pairing can take place in diverse combinations i.e., between lncRNAs, mRNAs, transposable elements (TE), and tRNA transcripts (Wang et al., 2005). Another class of secondary siRNAs known as *trans*-acting siRNAs (ta-siRNAs) are produced from non-coding *TAS* transcripts. ta-siRNA production is initiated by miRNA assisted cleavage of *TAS* transcripts, subsequent dsRNA synthesis and phased processing to produce siRNAs in a specific head to tail arrangement. Another sRNA class, the phasiRNAs are similar to ta-siRNAs and also produced in a phased manner, but ta-siRNAs are able to act only in *trans* (Fei et al., 2013; Yuan et al., 2015). The recently discovered class of 21 nt epigenetically activated siRNAs (ea-siRNAs) were found to be expressed from transposon-encoded transcripts in the *Decreased DNA Methylation 1 (DDM1)* mutant of *Arabidopsis.* These siRNAs are important to reduce or prevent transcription of TE-encoded RNAs and certain mRNA transcripts via siRNA-mediated silencing (Creasey et al., 2014). A TE-derived lncRNA (TE-lincRNA1195) was also reported to be involved in the ABA response and found to be important for abiotic stress adaptation (Wang et al., 2017).

Transcriptome analyses including mRNA and sRNA sequencing that provide insights into sRNA-based gene regulation in response to high light have not been performed in *Arabidopsis*. In our study, we performed transcriptome sequencing to identify the high light responsive ncRNA repertoire in *Arabidopsis* and analyzed its regulatory impact on associated target mRNA transcripts. We sequenced mRNA as well as sRNA libraries from *Arabidopsis* plants treated with high light acclimation conditions for 3 h, 6 h, and 2 days and investigated putative correlations between differentially expressed sRNAs from all classes and their cognate target RNAs. We identified a large number of sRNAs belonging to all known sRNA classes which were differentially expressed during the high light treatment and these sRNAs are able to control a large set target RNAs. Most of these targets encode transcription factors pointing to their role in modulation of gene expression.

## Materials and Methods

### Plant Material and Stress Treatment

Seeds of *A. thaliana* ecotype Columbia (*Col-0*), purchased from Nottingham Arabidopsis Stock Centre (NASC; United Kingdom), were sown at a high density (ca. 50 seeds on 9 × 9 cm pots) on a soil substrate and stratified for 2 days in the dark at 4°C. The pots were transferred into the light (LED-41 HIL2 cabinets, Percival, Perry, United States) following stratification and cultivated under control conditions with a light/dark regime of 16 h light (80 μmol photons m^–2^ s^–1^; corresponding to 18% of blue and red channel) at 22°C followed by 8 h dark at 18°C for 14 days. Plants serving as controls remained under these conditions whereas plants subjected to high light treatment were transferred 4 h after the onset of light at 22°C with a light intensity of 450 μmol photons m^–2^ s^–1^. A previous study reported that plants exposed to 450 μmol photons m^–2^ s^–1^ exhibited increased photoinhibition. The changes were found to be completely reversible during the de-acclimation phase, making this intensity of light the most suitable to study the high light acclimation related changes (Garcia-Molina et al., 2020). We used three biological replicates of control samples as well as the high light treated samples and harvested their aerial tissues after 3, 6, and 48 h. All the controls and their respective high light treated samples were processed at the same time. The high light treatment subjected to three subsequent biological replicates was performed in the same chamber with identical settings.

### RNA Isolation and Sequencing

The mRNA and sRNA sequencing data of untreated control samples obtained after 3 h, 6 h, and 2 days time points have been already published in our previous study (Tiwari et al., 2020). The mRNA sequencing data of high light treated biological triplicates obtained after 3 h and 2 days have been reported previously (Garcia-Molina et al., 2020). The samples for sRNA sequencing after 3 h, 6 h, and 2 days time points were processed together. Total RNA was isolated using TRI-Reagent (Sigma) following manufacturer’s protocol. Library preparations and sequencing were performed according to our previous study (Tiwari et al., 2020). Briefly, the mRNA/lncRNA libraries were prepared using the Next Ultra RNA Library Prep Kit (NEB) and were sequenced strand-specifically as 150 bp paired-ends with at least 15 million read pairs per library on Illumina HiSeq-2500 platform. sRNA libraries were prepared from 50 μg of total RNA using the NEBNext Multiplex sRNA Library Prep Kit (NEB)for Illumina following manufacturer’s instructions. The sRNA libraries were sequenced as 50 bp read length with a minimum of 7 million reads per library on Illumina HiSeq 1500.

### Bioinformatic Analyses of Transcriptomes

The 3 h, 6 h, and 2 days high light-acclimated samples along with their respective controls were sequenced and the mRNA/lncRNA sequencing data was analyzed using open web based platform GALAXY^1^ (Afgan et al., 2016). The adapter sequences were trimmed by FASTQ Trimmomatic tool using the default parameters. The Tophat tool mapped the raw reads against the *A. thaliana* TAIR10 reference genome^2^ with a maximum intron length parameter of 3,000 nt. The annotation of coding and non-coding RNA transcripts (≥200 bp) was performed using Araport11 annotation (Cheng et al., 2017). The FeatureCounts tool counted the number of reads mapped to the reference genome. The final list of genes was obtained by DeSeq2 tool of GALAXY using output from the FeatureCounts tool and classified using Araport11 reference annotation^3^.

The TAIR10 reference genome was used to map sRNA raw reads using the Shortstack software (Axtell, 2013). Approximately 80% of the obtained reads efficiently mapped to the reference. A reference annotation database was created from publicly available sources such as miRNA (miRBase version 22.1), lncRNA (Araport11), *trans-* and *cis-*nat-siRNA, ta-siRNA, and phasiRNA (Howell et al., 2007; Jin et al., 2008; Zhang et al., 2012; Yuan et al., 2015). Using these sources, the read counts of different classes of sRNAs were calculated. The read counts of the triplicates of samples were later analyzed by the DeSeq2 tool and differentially expressed (DE) sRNAs with FC ≥ 2 and ≤−2 (Benjamini-Hochberg corrected *p*-value ≤ 0.05) were identified.

### Prediction of Putative miRNA Targets

psRNATarget: A Plant Small RNA Target Analysis Server (2017 Update) was used to identify putative miRNA targets (Dai et al., 2018). DE miRNAs were used as a query to search against *A. thaliana* Araport11 transcript database using default parameters with changes in UPE (25) and maximum expectation (2.5) to ensure more stringent predictions.

### Gene Ontology Analyses of Putative miRNA Targets

We used the DAVID Bioinformatics tool (Jiao et al., 2012) to perform the GO analyses. The list of miRNA target genes was provided as an input and the output gene list was broadly classified into biological process, cellular compartment and molecular function. The significant GO terms were identified in all aforementioned categories (Fisher’s test with Benjamini-Hochberg corrected *p*-value ≤ 0.05). The ggplot2 package^4^ was used for visualization of the output.

### cDNA Synthesis for Stem Loop qRT-PCR

We used 300 ng of RNA from three biological replicates of treated and untreated samples as the starting material for cDNA synthesis. DNAse I (2 U, NEB) was added to the RNA and incubated at 37°C for 30 min to eliminate genomic DNA contamination, and later was inactivated at 65°C for 10 min. Reverse transcription of RNA into cDNA was performed by M-MuLV Reverse transcriptase (200 U, NEB) at 42°C for 30 min. The stem loop primers specific for the sRNAs and a universal reverse primer were used for cDNA synthesis (Supplementary Table 1). We used *UBI1* (*AT4G36800*) specific reverse primer during the reverse transcription and confirmed successful cDNA synthesis through RT-PCR by using *UBI1* specific gene primers.

### Stem Loop qRT-PCR

We performed qRT-PCR using EvaGreen and sRNA-specific primers (Kramer, 2011; Supplementary Table 1). The qRT-PCRs were performed in three technical replicates for each sample and each reaction contained cDNA amounts equivalent to 20 ng/μl of initial RNA. The qRT-PCR program was subjected to initial denaturation at 95°C for 2 min followed by 40 cycles of amplification with 95°C for 12s, annealing for 30s and 72°C for 15s. After each cycle, the EvaGreen signals were measured and melting curves were monitored to confirm primer specificities. The ΔΔCt method was used to calculate the expression levels following normalization against *UBI1* housekeeping gene (Habermann et al., 2020; Tiwari et al., 2020).

## Results

### Changes in the sRNA Repertoire During High Light Acclimation in *A. thaliana*

*A. thaliana* seedlings were subjected to high light treatments (450 μmol photons m^–2^ s^–1^ for 3 h, 6 h, and 2 days) to analyze high light-responsive changes in the sRNA repertoire. A previous study related to high light acclimation observed marked mRNA expression changes after 3 h and 2 days time points (Garcia-Molina et al., 2020). An additional early time point was included i.e., 6 h to address regulation of gene transcripts as miRNAs are known to control early changes in gene expression (Barciszewska-Pacak et al., 2015; Patel et al., 2019). A minimum of 7 million reads per library of treated and control samples were generated for transcriptome profiling. For all samples, mapping of sRNA reads against the *A. thaliana* reference genome revealed an average of about 13% reads mapping to miRNA loci, 10% to *trans-* and 2% to *cis*-nat-siRNA loci. 5% of the remaining reads corresponded to lncRNAs, 3% to ta-siRNA producing regions and 0.3% to phasiRNAs (Supplementary Tables 2, 3). Only approximately 1% of the reads accounted for loci encoding the most abundant RNAs such as rRNA, snoRNA, tRNA, and snRNA validating high quality of the sRNA libraries. The remaining reads mostly mapped to other RNA classes involved in epigenetic regulations such as TE and repeat associated regions.

**FIGURE 1:**
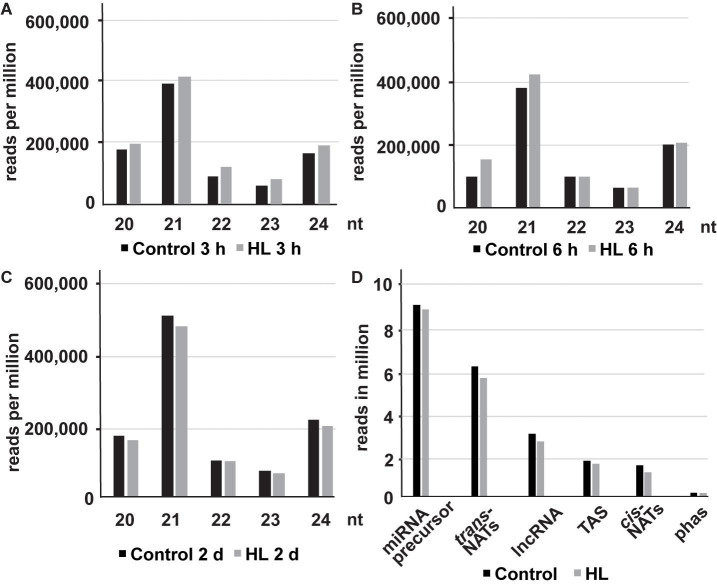
The size distribution (20–24 nt) of mapped sRNAs after 3 h **(A)**, 6 h **(B)** and 2 days **(C)** of high light treatment (represented in reads per million). The distribution of trimmed ncRNA reads from different RNA classes (reads per million) in untreated and high light acclimated samples **(D)**.

The size distribution of sRNAs showed two distinct peaks at 21 and 24 nt. The peak at 21 nt indicates an enrichment of miRNAs, nat-siRNAs, and ta-siRNA whereas the peak at 24 nt represents sRNAs derived from repetitive/intergenic RNAs, inverted repeats, and TE (Figures 1A–C and Supplementary Table 4). We observed an increased sRNA abundance at the early time points (3 and 6 h) and reduced production after 2 days compared to the control samples in response to high light acclimation. Our analyses revealed that miRNAs and *trans*-nat-siRNAs are the two major sRNA classes uncovered in our data set (Figure 1D).

**FIGURE 2:**
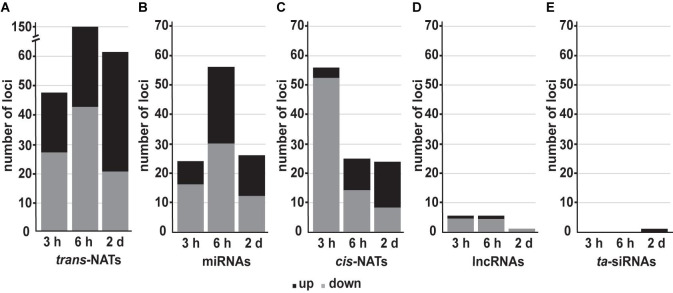
Number of detected sRNAs belonging to different sRNA classes during high light acclimation. The number of up- (black) and downregulated (gray) sRNAs from *trans*-NATs **(A)**, miRNAs **(B)**, *cis*-NATs **(C)**, lncRNAs **(D)**, and ta-siRNAs **(E)** in response to high light treatment after 3 h, 6 h, and 2 days (FC ≥ 2 and ≤−2, Benjamini-Hochberg corrected *p*-value ≤ 0.05).

To identify DE sRNAs between treated samples and the controls, the values of normalized reads were used to calculate the relative expression of mature miRNAs and siRNAs (FC ≥ 2 and ≤−2, Benjamini-Hochberg corrected *p*-value ≤ 0.05). Over the analyzed time points, high light affected sRNAs were mainly generated from *trans*- followed by *cis*-NAT-pairs and miRNAs (Figures 2A–C). We observed an increasing number of upregulated *trans*-nat-siRNAs and *cis*-nat-siRNAs over the time course of high light treatment (Figures 2A,C). The differential expression of miRNAs substantially increased after 6 h of treatment (Figure 2B). Differentially expressed sRNAs from lncRNAs (Figure 2D) and ta-siRNAs (Figure 2E) were the least in count and most of the lncRNA derived sRNAs were downregulated.

**FIGURE 3:**
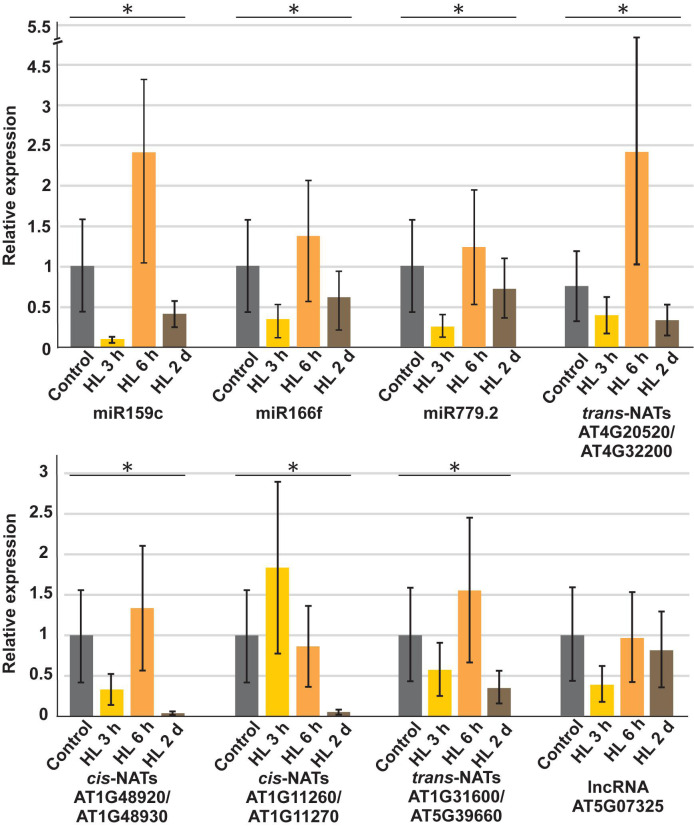
Stem loop qRT-PCR based validation of sRNA sequencing data for miRNAs as well as sRNAs derived from *trans*-NATs, *cis*-NATs and lncRNA. Expression values are normalized to *UBI1* housekeeping gene and the untreated control was set to 1. The error bars indicate the standard deviation (*n* = 3). Statistically significant differences for each sRNA were analyzed by one-way ANOVA (*p* < 0.05) and represented by an asterisk.

To confirm the reliability and validity of our sRNA sequencing data, we performed stem loop RT-PCRs to determine the steady-state levels for selected sRNAs produced from all analyzed RNA classes during the course of high light treatment (Figure 3). Statistically significant differences for each sRNA were analyzed by one-way ANOVA and other than lncRNA *AT5G07325*, changes in relative expression levels were significantly different (*p* < 0.05). miR159c, miR166f, miR779.2, *trans*-nat-siRNAs produced from *AT4G20520-AT4G32200*, and *AT1G31600-AT5G39660* transcripts, *cis*-nat-siRNAs derived from *AT1G48920-AT1G48930* were differentially expressed upon high light treatment and *AT1G11260-AT1G11270* were repressed after 6 h, whereas sRNAs derived from lncRNA *AT5G07325* were downregulated after 3 h of high light treatment confirming our sequencing results.

### Expression Profiling of miRNAs During High Light Acclimation

Next generation sequencing distinguishes between individual miRNAs even with a single nucleotide polymorphism and obtained reads were analyzed to determine differentially regulated miRNAs (FC ≥ 2 and ≤-2, Benjamini-Hochberg corrected *p*-value ≤ 0.05) after precise read mapping (Table 1 and Supplementary Table 5). We observed a general trend in all samples that around 11% of the detected miRNAs possessed very high normalized read counts (>1,000 reads per sample), about 38% showed moderate expression (<1,000 and >20 normalized reads), 15% showed reduced read counts (<20 and >5 normalized reads) and 36% showed very low expression (<5 normalized reads) (Supplementary Table 6). In response to high light treatment, we observed 24 DE miRNAs (8 up and 16 down) after 3 h, 56 mature DE miRNAs (26 up and 30 down) after 6 h and 26 DE mature miRNAs (14 up and 12 down) after 2 days (Supplementary Table 5).

**TABLE 1:** Putative miRNA:mRNA target pairs and their relative expression patterns upon 3 h, 6 h, and 2 days of high light treatments.

miRNA:mRNA pairs	3 h	6 h	2 days
↑↓	3	6	2
↑↑	0	4	5
↓↓	3	1	1
↑− or ↓−	18	34	36
↑∘ or ↓∘	104	253	131

*The first arrow corresponds to miRNA regulation and the second to the regulation of its target mRNA transcripts and the arrows represent the correlation expression as follows: ↑ = upregulated, ↓ = downregulated, − = unchanged, ∘ = undetected.*

Conserved miRNA families seem to have important functions since they mainly regulate targets encoding TFs or enzymes acting in abiotic stress adaptation (Qin et al., 2014; Khaksefidi et al., 2015; Yu et al., 2019). Over the last few years, 22 miRNA families were identified to be conserved between *A. thaliana*, *Oryza sativa and Populus trichocarpa* (Bonnet et al., 2004; Zhang et al., 2006; Pelaez et al., 2012). Out of these 22 miRNA families, members of 16 families were found to be differentially expressed upon high light; corresponding to 8, 11, and 13 DE mature miRNAs at 3 h, 6 h, and 2 days, respectively (Supplementary Table 7). It is known that miRNAs regulate the expression of TFs and are involved in phyB-mediated light signaling pathways and there are very few light responsive miRNAs identified in crop plants (Sun et al., 2015; Yang et al., 2019). In total, 92 non-redundant mature miRNAs were found to be differentially expressed throughout the course of high light treatment (Supplementary Table 7). Out of these 92 mature miRNAs, 38 mature miRNAs belonging to 14 conserved miRNA families are light stress regulated in other plant species (Casati, 2013) and 46 mature miRNAs have been previously known to be UV-B, white light, and high light responsive in *A. thaliana* (Supplementary Table 7). Our study shows similarity in the induction or repression pattern of these miRNAs compared to other light stress-related studies (Zhou et al., 2007; Shikata et al., 2014). The remaining 46 DE mature miRNAs belonging to 37 miRNA families such as miR447, miR861 and miR863 have not been reported before to be light-regulated in *A. thaliana* (Supplementary Table 7). We identified 5 miRNAs with a varying expression pattern, i.e., up- and downregulation, and 7 miRNAs with consistent expression pattern (either up- or downregulated) in at least two of the analyzed time points. We found miR399a to be consistently upregulated at all the three time points. This miRNA was also found to be upregulated by red light in leaves of potatoes and in phosphorous deficient conditions in barley (Hackenberg et al., 2013; Qiao et al., 2017).

### Differentially Expressed miRNA Targets

MiRNAs can mediate the cleavage of their mRNA targets or cause translation inhibition (Aukerman and Sakai, 2003). Plant miRNAs show perfect or partial sequence complementarity to their target sequences and often lead to mRNA cleavage between nucleotides 10 and 11 of the miRNA binding site (Bartel, 2004; Brodersen et al., 2008). We sequenced the sRNAs and mRNA/lncRNA from the same RNA samples and directly compared changes in miRNA expression with the changes of their cognate targets. We used the psRNATarget analysis server with stringent search criteria to determine the targets of DE miRNAs during the time course of high light treatment (Dai et al., 2018) and found putative targets for 88 of 92 DE miRNAs comprising 322 mRNAs and 15 ncRNAs (Supplementary Tables 8, 9). The 19 DE miRNAs (8 up- and 11 downregulated) at 3 h of high light acclimation can target 100 non-redundant mRNAs and 3 non-coding transcripts. The 50 DE miRNAs (23 up- and 27 downregulated) at 6 h of treatment are able to target 220 non-redundant mRNAs and 10 non-coding RNA targets and the 25 DE miRNAs (14 up- and 11 downregulated) after 2 days can target 125 non-redundant mRNAs and 5 non-coding RNA targets (Supplementary Tables 8, 9).

On the basis of Araport annotation (V11; see text footnote 3; Cheng et al., 2017), we identified 30 targets of DE miRNAs from all the five subgroups to be consistently present throughout the course of high light treatment (Supplementary Table 10). These mRNAs mainly encode transcription factors and integral membrane proteins. We also examined the putative function of miRNA targets that were specifically observed in each time point. At the 6 h time point we found several pentatricopeptide repeat proteins (PPR), important for RNA maturation in various organelles, tetratricopeptide repeat (TPR) proteins acting in signaling and organellar import, and S-adenosyl-L-methionine-dependent methyltransferases superfamily proteins, necessary for epigenetic regulation of gene expression. At the 2 days time point we detected transcripts encoding auxin response factors, GRAS family transcription factors and MYB domain proteins that are involved in transcriptional regulation in response to stress.

### Target Prediction for Differentially Expressed miRNAs

To investigate how the regulation of these putative targets correlates with the changes in the miRNA repertoire, our mRNA as well as lncRNA transcriptome data generated from the identical RNA pools were further analyzed (Supplementary Tables 11, 12). We investigated the correlation between the 88 DE miRNAs and their cognate 332 target transcripts (Supplementary Table 8). Even though we mainly observed that one transcript can be targeted by various isoforms of a miRNA family, we found few cases in which target transcripts can also be cleaved by different miRNAs that are not related in sequence. By taking all individual DE miRNAs and their cognate protein-coding transcripts (mRNAs) as miRNA:mRNA pairs into consideration, we identified 128, 302, and 175 miRNA:mRNA pairs for the 3 h, 6 h, and 2 days time points of high light treatment, respectively (Supplementary Tables 8). We broadly classified the miRNA:mRNA target pairs of all time points into different categories based on the correlation between miRNAs and their cognate mRNA expressions. These broad categories are (i) inverse correlation (when miRNA and mRNA show anticorrelation), (ii) same tendencies (when both miRNA and mRNA either upregulated or downregulated), (iii) steady (or undetected) levels of target mRNA despite changes in miRNA levels (Table 1). We observed 3, 6, and 2 inversely correlated pairs at 3 h, 6 h, and 2 days, respectively, with a total number of 10 non-redundant inversely correlated miRNA:mRNA target pairs that modulate the mRNA repertoire upon high light treatment (Supplementary Table 8). Apart from the mRNA targets, psRNATarget prediction server additionally predicted 17 putative non-coding RNA targets of DE miRNAs, but the expression levels of those ncRNA target transcripts were either unchanged or their levels were below detection limit (less than 5 reads).

We used available degradome datasets to analyze the overlap of previously known miRNA targets and the targets found in this study. Through the course of high light treatment, we observed 522 non-redundant miRNA:mRNA target pairs and out of these 167 pairs (∼32% of total pairs) have been found to be predicted or validated in at least one of the previous studies (Carrington data set)^5^ (Addo-Quaye et al., 2008; Ahmed et al., 2014; Brousse et al., 2014). We found 37 pairs out of 124, 98 out of 300, and 56 pairs out of 173 after 3 h, 6 h, and 2 days of treatment, respectively, to be previously validated or predicted (Supplementary Table 13). A high correspondence of previously known miRNA targets indicates reliability of our data.

We detected an inverse correlation between 10 miRNAs and their putative targets (Table 2), for example, after 3 h of high light treatment we noticed upregulation of miR864-3p (FC = 3.65) and downregulation of its novel predicted target *Dark inducible 4* (DIN4, FC = −2.31) which is known to be induced in darkness in *A. thaliana* (Fujiki et al., 2000) and suggests that miR864-3p represses *DIN4* expression in high light. At the same time point, we found miR172b-3p to be upregulated (FC = 2.47) and its novel putative target *Hydroxysteroid dehydrogenase 3* (*HSD3*, FC = −2.05) to be downregulated. It is known that plant cell membranes contain sterols that are synthesized by hydroxysteroid dehydrogenases/decarboxylases (Kim et al., 2012) and that light stress has an impact on the composition of sterols in the cell membranes (Kuczynska et al., 2019). After 6 h of high light treatment we observed upregulation of miR156d-5p (FC = 2.42) and a concomitant downregulation of its validated target transcript encoding the Squamosa promoter binding protein-like 3 (*SPL3*, FC = −2.14). A previous study has shown that constitutive expression of miR156 extended the transition from the juvenile to vegetative phase resulting in delayed flowering (Wu and Poethig, 2006). Thus, it is likely that high light leads to an upregulation of miR156 and a concomitant downregulation of *SPL3* to delay flowering. Another miRNA, miR171c-5p showed reduced expression levels (FC = −2.53) after 6 h of high light treatment whereas its novel predicted target encoding APS reductase 3 was upregulated (FC = 2.26). APS reductase is the key enzyme of sulfate assimilation and was previously reported to increase in response to sugar and light (Kopriva et al., 1999) suggesting a regulatory role of miR171c-5p in this process. Additionally, after 6 h and 2 days of high light treatment, we observed a downregulation of miR395a and an upregulation of its novel predicted target transcript encoding cellulose synthase like G3 which is responsible for producing the polysaccharide cellulose, the main component of the plant cell wall.

**TABLE 2:** List of miRNA:mRNA target pairs showing inversely correlated expression in response to high light treatment after 3 h, 6 h, and 2 days time points (miRNA and mRNA fold change ≥ 2 or≤−2, Benjamini-Hochberg corrected *p*-value ≤ 0.05).

miRNA	miRNA log_2_FC	Target	Description	mRNA log_2_FC	*p*-value	*q*-value
3 h						
Ath-miR169i	1.22	AT5G42120	Lectin receptor kinase S.6	-1.59	0.00	0.00
Ath-miR864-3p	1.87	AT3G13450	Dark inducible 4	-1.22	0.00	0.00
Ath-miR172b-3p	1.32	AT3G47360	Hydroxysteroid dehydrogenase 3	-1.05	0.01	0.04
6 h						
Ath-miR163	1.58	AT1G15125	S-adenosyl-L-methionine-dependent methyltransferases superfamily protein	-1.12	0.01	0.04
Ath-miR156d-5p	1.29	AT2G33810	Squamosa promoter binding protein-like 3	-1.10	0.00	0.01
Ath-miR395a	-5.48	AT4G23990	Cellulose synthase like G3	1.46	0.00	0.00
Ath-miR171c-5p	-1.35	AT4G21990	APS reductase 3	1.19	0.00	0.00
Ath-miR831-3p	-1.53	AT3G11120	Ribosomal protein L41 family	1.10	0.00	0.00
Ath-miR158a-5p	-1.65	AT5G27395	Mitochondrial inner membrane translocase complex, subunit Tim44-related protein	1.03	0.00	0.00
2 Days						
Ath-miR395a	-1.44	AT4G23990	Cellulose synthase like G3	1.23	0.00	0.00
Ath-miR168a-3p	-1.07	AT3G07195	RPM1-interacting protein 4 family protein	1.11	0.01	0.07

### Gene Ontology Analysis of Predicted miRNA Targets

**FIGURE 4:**
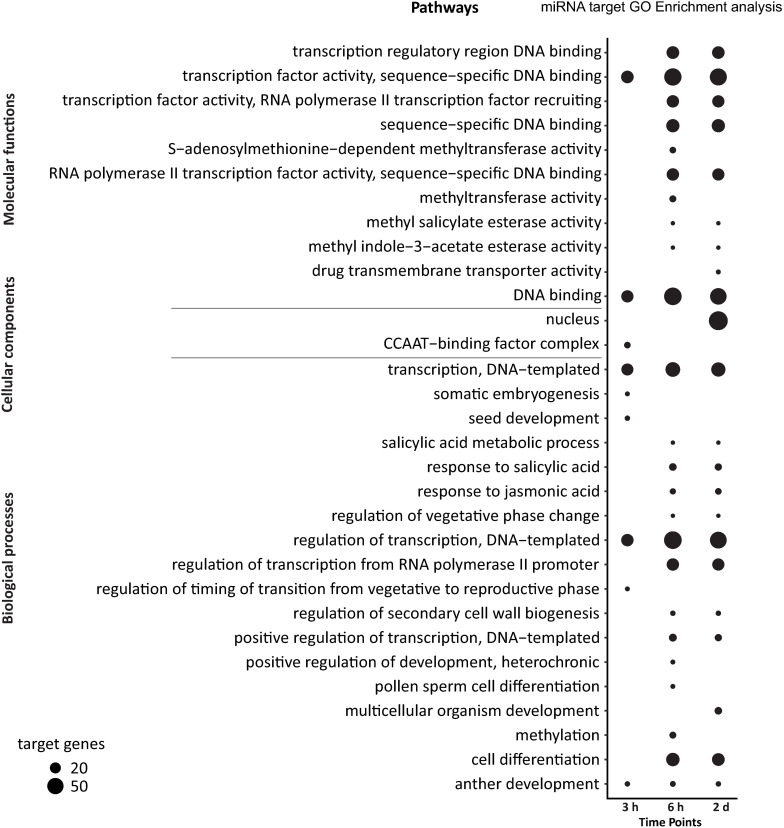
Gene ontology analysis for all predicted targets of DE miRNAs in high light acclimation. The dot plot represents GO terms categorized into molecular functions, cellular components and biological processes. The GO terms and the time points of the high light treatment were depicted on the *y*- and *x*-axis, respectively. The bubble size represents the number of genes in that particular GO term (Benjamini-Hochberg corrected *p*-value ≤ 0.05).

We used the David bioinformatics tool (Jiao et al., 2012) to perform gene ontology (GO) analysis for the putative targets of DE high light responsive miRNAs to obtain information about the possible role of the targets. Based on the three categories of GO biological processes, cellular component and molecular function, an enrichment of GO terms for all time points was observed (Fisher’s test with Benjamini-Hochberg corrected *p*-values) (Figure 4 and Supplementary Table 14). After 3 h time point the significant biological processes included regulation of transcription (32) and transcription (30). Within the cellular component category, the highest number of targets were associated with the CCAAT-binding factor complex (8). Furthermore, in the molecular functions category, regulatory proteins involved in gene transcription such as TF activity, sequence-specific DNA binding (33) and DNA binding (31) were significantly overrepresented. After 6 h of high light treatment, miRNA targets were mainly involved in regulation of transcription (68), cell differentiation (38), salicylic acid response (11), methylation (9) and jasmonic acid response (7), and similar to the 3 h time point indicating an enrichment of genes associated with transcriptional control. We also found methyltransferase activity (9) to be significantly enriched in the category molecular function suggesting epigenetic modifications and a potential role in secondary cell wall biogenesis. At the 2 days time point, we detected an enrichment of significant biological processes including regulation of transcription (61), cell differentiation (34), multicellular organism development (11) and jasmonic acid response (8). Thus, at all the three time points, genes encoding proteins involved in transcriptional reprogramming upon high light acclimation were enriched. The category cellular components showed a striking enrichment of targets associated with the nucleus (78 target genes) nicely matching the enrichment of transcription-related biological processes and molecular functions that points to massive changes in transcriptional regulation in response to high light acclimation (Figure 4).

### sRNAs Derived From Non-overlapping lncRNAs

The sRNA sequencing data was used to analyze miRNA regulation as well as to identify sRNAs derived from other RNA classes in response to high light and to prove their roles in high light acclimation. After mapping the sRNA reads against publicly available reference databases (Jin et al., 2008; Zhang et al., 2012; Wang H. et al., 2014; Yuan et al., 2015), we revealed a high number of DE sRNAs associated to lncRNAs, *trans-* and *cis-*NATs pairs, *TAS* and *PHAS* RNAs.

Non-overlapping lncRNA transcripts, ≥200 nt in size, do not overlap with protein encoding or other non-coding transcripts. In our sRNA data 11 non-redundant non-overlapping lncRNA loci which produce DE sRNAs were determined and two of these 11 lncRNA loci upregulated sRNAs whereas the remaining 11 downregulated sRNAs upon high light (Supplementary Table 15). The transcript levels of the lncRNAs remained unchanged across all analyzed samples, but we observed DE sRNAs generated from these lncRNAs. We found 5, 5, and 1 lncRNA at 3 h, 6 h, and 2 days time point after high light treatment, respectively, that produced DE sRNAs. We found differentially expressed 24 nt sRNAs derived from lncRNA *AT4G05135*, and *AT3G05925* and 21 nt sRNAs produced from lncRNA *AT5G07325* after 3 h time point. At 6 h time point, lncRNA *AT3G26612*, *AT3G04485*, and *AT4G04965* gave rise to differentially expressed 24 nt sRNAs and *AT5G04445* produced increased 21 nt sRNAs. We found one ncRNA *AT1G06797* that generated reduced levels of 24 nt sRNAs after 2 days of treatment. There were 4 sense strand (AT2G14878, AT5G04445, AT3G26612, and AT5G06045) and 3 antisense strand lncRNA transcripts (AT5G07565, AT5G07325, and AT4G04965) that produced DE sRNAs, and strand specificity was undetected for the remaining 4 lncRNA loci. Furthermore, since the lncRNAs do not overlap with any other gene and do not have any trans pairing partner, we speculate that the sense strand lncRNAs are converted into dsRNA by RNA dependent RNA polymerases in a primer independent manner. The lncRNA antisense transcripts also have a capability to form stem-loop fold back structures which can produce sRNAs.

### sRNAs Derived From Natural Antisense Transcripts

The NAT pairs can form dsRNAs due to sequence complementarity and can arise from overlapping non-coding (nc) or protein coding (pc) genes. The transcript pairing is possible between pc-pc, nc-pc and nc-nc transcripts and the resulting paired transcript can be targeted by DCL enzymes to produce nat-siRNAs. The majority of *cis-* and *trans*-NAT pairs were produced from pc:pc or pc:nc transcript pairs. In case of pc:nc, the nc pairing partner mostly represents tRNA or TE derived transcripts which also have the capacity to produce sRNAs individually (Creasey et al., 2014; Martinez et al., 2017; Cho, 2018). The pre-tRNA and TE-derived sRNAs could contribute to the regulation of a high light acclimation related network by regulating their own as well as other transcripts by sequence complementarity (Loss-Morais et al., 2013; Cho, 2018). We revealed that transcript pairs producing elevated levels of sRNAs can have different expression patterns. We observed abundant transcript pairs that generate differentially expressed nat-siRNAs, but the transcripts were either undetected or unchanged in the mRNA data. We further identified pairs of transcripts where one transcript is regulated and the other remains unchanged, anticorrelated pairs with one transcript up- and the other transcript downregulated, and pairs showing the same changes in expression (both transcripts either upregulated or down regulated).

### *Cis*-nat-siRNAs

We found 56, 25, and 24 *cis*-NATs loci (90 non-redundant pairs) at 3 h, 6 h, and 2 days, respectively, that produced DE *cis*-nat-siRNAs from two overlapping transcripts. We detected 7, 3, and 2 loci at 3 h, 6 h, and 2 days, respectively, where one of the transcripts was either up- or downregulated and the other one remained unchanged (Supplementary Table 16). At 3 h time point, we observed that all the 7 loci encoding pc:pc transcripts reduced the production of sRNAs with at least twofold decrease in one of their parent transcripts. The decrease in one of the parent transcripts and thus the sRNAs could be due to transient changes in response to high light stress (Supplementary Table 16). We detected 49, 21, and 20 *cis*-NATs (76 non-redundant pairs) at 3 h, 6 h, and 2 days time point, respectively, that produced DE sRNAs from cognate overlapping transcripts that remained unchanged or were undetectable (Supplementary Table 16). At the 6 h time point, we observed upregulation of sRNAs from a *cis*-NAT transcript pair where one of the pairing transcripts encoding NUCLEOLIN LIKE 1 was upregulated. This gene was also shown to be upregulated by salt stress and to play a role in ribosome biogenesis (Huang et al., 2018). We detected another transcript pair with reduced nat-siRNA production where one transcript encoding the ARABIDOPSIS HOMOLOGUE OF YEAST BRX1-1 (AT3G15460) was upregulated and the other transcript encoding an aluminum induced protein with YGL and LRDR motifs (AT3G15450) was downregulated. It has been shown that AT3G15450 is regulated by ABA since an ABA hypersensitive mutant (*ahg2-1*) shows reduced levels of this gene in response to high light stress (Nishimura et al., 2005; Valdivieso et al., 2009). After 2 days of high light stress, we found two gene pairs generating elevated levels of nat-siRNAs where the pairing transcripts were also upregulated. In each pair, one transcript encodes a lncRNA (AT3G51238 and AT5G01595) and the other encodes flavanone 3-hydroxylase (AT3G51240) and FERRETIN 1 (AT5G01600), respectively. The upregulation of *flavanone 3-hydroxylase* is associated to the biosynthesis of flavonoids where it catalyzes the conversion of flavanones to dihydroflavonols whereas *FERRETIN 1* plays a role in iron homeostasis (Pelletier and Shirley, 1996; Briat et al., 2010). At the same time point, we found upregulated nat-siRNAs derived from two gene pairs where one transcript was upregulated and the other remained unchanged. Interestingly, the upregulated transcripts of these pairs encode for chalcone synthase (AT5G13930) known to be the rate-limiting enzyme involved in flavonoid synthesis (Fuglevand et al., 1996) and a MULTIDRUG RESISTANCE-ASSOCIATED PROTEIN 2 (AT2G34660) which was shown to assist in vacuolar transport of anthocyanins and flavonoids (Behrens et al., 2019). This suggests a possible involvement of nat-siRNAs in regulation of these transcripts in biosynthesis and transport of flavonoids which could play a major role in protection of plant against high light stress.

### *Trans*-nat-siRNAs

We found 17 non-redundant *trans*-NAT pairs (0, 12, and 5 at 3 h, 6 h, and 2 days, respectively) producing differentially expressed *trans*-nat-siRNA. In this case, transcripts can produce sRNAs from their overlapping region or from the single stranded region when partially overlapped. We observed 40, 124, and 52 (84 non-redundant loci) *trans*-NATs gene pairs at 3 h, 6 h, and 2 days, respectively, that promote DE *trans*-nat-siRNAs from the overlapping region of two transcripts having unchanged transcript levels or levels below the detection limit (Supplementary Table 17). We observed 3, 5, and 3 *trans*-NAT pairs comprising overlapping pc:pc transcripts that generate DE *trans*-nat-siRNAs. The majority of the *trans*-NAT gene pairs comprise a nc transcript partner encoding a pre-tRNA or RNA derived from a TE. Apart from the pc:pc pairs at 3 h, 6 h, and 2 days time point, we found 34, 124, and 44 transcript pairs generating DE *trans*-nat-siRNAs which are comprised of one transcript encoding a pre-tRNA and 7, 13, and 4 pairs where one of the transcripts is encoded by a TE transcript. The profiling of *trans*-nat-siRNAs over time revealed that the highest number of DE *trans*-nat-siRNAs were found after 6 h proposing the involvement of *trans*-nat-siRNA in modulating gene expression during early stages of high light acclimation.

### ta-siRNAs

After 2 days of high light treatment, we observed an upregulation of ta-siRNAs derived from the *TAS4* precursor (Supplementary Table 18) that requires miR828-mediated cleavage prior to ta-siRNA biogenesis (Rajagopalan et al., 2006).

**FIGURE 5:**
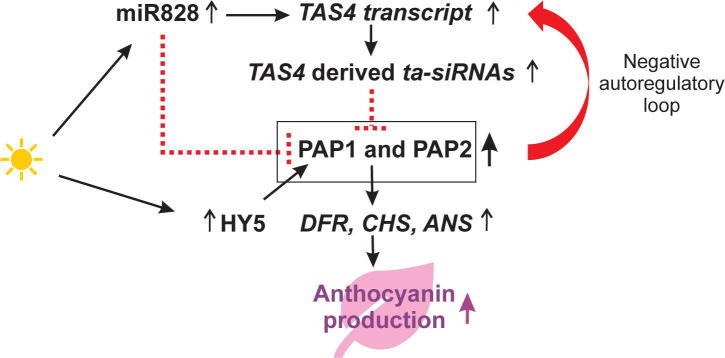
Our current model on the regulation of anthocyanin biosynthesis in response to high light. Increased PRODUCTION OF ANTHOCYANIN PIGMENT 1 (PAP1) can bind to the PAP1 *cis*-elements of the *TAS4* gene to induce *TAS4* transcription. In response to high light, miR828 is upregulated and triggers the production of *TAS4* derived ta-siRNAs. The ta-siRNAs can target *PAP1* and *PAP2* mRNAs and downregulate their transcript levels. Additionally, high light induced miR828 can also downregulate *PAP1* transcripts. Furthermore, high light induces the transcription factor ELONGATED HYPOCOTYL 5 (HY5) that binds to the *PAP1* promoter and activates *PAP1* transcription that in turn provokes transcription of genes encoding enzymes involved in anthocyanin biosynthesis such as DIHYDROFLAVONOL 4-REDUCTASE (DFR), CHALCONE SYNTHASE (CHS) and ANTHOCYANIDIN SYNTHASE (ANS). After 2 days of high light treatment, the concomitant increase in the components of the proposed model were confirmed by our mRNA sequencing data which support previous findings (Hsieh et al., 2009; Luo et al., 2012; Yang et al., 2013). Black arrows symbolize positive regulation and red dashed lines indicate negative regulation.

In response to sugar accumulation, *TAS4* expression is regulated through a signaling pathway involving PRODUCTION OF ANTHOCYANIN PIGMENT 1 (PAP1) (Luo et al., 2012). The *TAS4* derived ta-siRNAs are capable of targeting mRNAs encoding MYB transcription factors such as PAP1 and PAP2 which regulate the anthocyanin biosynthesis pathway. *MIR828* overexpression lines showed reduced anthocyanin accumulation since miR828 is also known to target *PAP1* (Yang et al., 2013). We found increasing levels of miR828 (FC = 20.9), *TAS4* transcript (AT3G25795, FC = 3.27), *ta-siRNAs* (FC = 6.32), *PAP1* (AT1G56650, FC = 5.20), *PAP2* (AT1G66390, FC = 6.77), and ELONGATED HYPOCOTYL 5 (*HY5*, AT5G11260, FC = 1.67) that all play a role in anthocyanin biosynthesis. In addition to the altered expression of these regulators, we also found increased amounts of downstream anthocyanin biosynthetic enzymes i.e., DIHYDROFLAVONOL 4-REDUCTASE (*DFR*, AT5G42800, FC = 12.1), CHALCONE SYNTHASE (*CHS*, AT5G13930, FC = 5.16) and ANTHOCYANIDIN SYNTHASE (*ANS*, AT4G22880, FC = 14.7) (see also discussion, Figure 5). According to our sequencing data, we can provide evidence that in response to high light stress, the *PAP1* transcript is negatively regulated by miR828, but positively regulated by the transcription factor HY5 (Shin et al., 2013) thereby suggesting a negative autoregulatory loop between PAP1-TAS4 and ta-siRNAs. The consequent increase in the components of the anthocyanin biosynthetic pathway is likely to maintain the increased levels of anthocyanin production required to protect plants from high light.

## Discussion

Transcriptome studies by mRNA and sRNA sequencing in response to high light stress and white light, respectively, have been conducted in several plant species, whereas a global transcriptome analysis of sRNAs in response to high light acclimation has not been performed yet. Our study aims to provide insights into the high light-responsive regulation of different classes of sRNAs and their effects on the modulation of gene expression. We performed sRNA sequencing along with mRNA and lncRNA sequencing from the same RNA samples in order to associate changes in target transcripts (mRNA/lncRNA) and changes in sRNA repertoire upon high light. Over the time course of high light treatment, the number of DE sRNAs from all different classes showed a gradual increase during the early stages (3 h and 6 h) of high light treatment and a reduction after 2 days (Supplementary Table 5). We analyzed the miRNAs which are known to be important regulators of gene expression in eukaryotes and detected 92 DE miRNAs over the course of high light treatment and out of these, 44 DE miRNAs were shown before to be responsive to UV-B, white light or high light in *A. thaliana* (Supplementary Table 7; Bonnet et al., 2004; Zhang et al., 2006; Zhou et al., 2007; Shikata et al., 2014). To determine the impact of DE miRNAs on the transcriptome of *A. thaliana* we investigated their targets predicted by the psRNATarget tool and found 128, 302, and 175 potential miRNA:mRNA target pairs at 3 h, 6 h, and 2 days time point, respectively (Supplementary Table 8). The high number of putative miRNA targets at the early time points reflect the importance of miRNAs in regulating the gene expression at the initial stages of the high light treatment. At early time points, we observed targets encoding PPR and TPR proteins which could lead to alterations in the process of RNA maturation, stress signaling and organellar transport. The miRNA targets also include members of the S-adenosyl-L-methionine-dependent methyltransferase superfamily proteins indicating a possible epigenetic regulation of gene expression in response to high light.

GO analysis revealed a large number of putative targets encoding transcription factors such as MYB, squamosa promoter binding proteins (SPBs), Teosinte Branched 1, Cycloidea, members of the PCF (TCP) TF family and members of the Homeodomain-like superfamily (Supplementary Table 14). The enrichment of these TFs clearly indicates their involvement in high light-induced regulation of gene expression. Studies on high light and salinity stress in *A. thaliana* have shown that MYB TFs are principal regulators of flavonoid biosynthesis. MYB112 was found to be induced by high light stress and to regulate anthocyanin biosynthesis. It can be hypothesized that the *MYB* transcripts targeted by miRNAs in our study are directly or indirectly involved in the regulation of anthocyanin biosynthesis to protect the plant against high light. We observed enrichment of genes responsive to phytohormones such as jasmonic acid (JA) and salicylic acid (SA) which are known to crosstalk in order to induce high light acclimation (Mateo et al., 2006; Balfagon et al., 2019). Studies in wheat and barley subjected to UV-B stress in the presence of exogenous JA reported an increased antioxidant signaling, enhanced proline levels and elevated ROS scavenging capabilities (Fedina et al., 2009; Liu et al., 2012). Similarly, the role of SA in response to high light and its role in redox homeostasis was elucidated in *A. thaliana* (Mateo et al., 2006). After 6 h of high light treatment, we observed an upregulation of miR156d-5p and a concomitant downregulation of its target *SPL3* (Supplementary Table 8). miR156-SPLs affect the anthocyanin biosynthesis pathway and control development in stress conditions (Cui et al., 2014). Constitutive overexpression of miR156 caused an extended juvenile phase and delayed flowering (Wu and Poethig, 2006). It was shown that flowering promoting transcription factors such as *LEAFY* (*LFY*), *FRUITFULL* (*FUL*), and *APETALA1* (*AP1*) increased in levels due to upregulation of *SPL3* (Yamaguchi et al., 2009). Our results suggest that high light causes upregulation of miR156 that mediates downregulation of its cognate target *SPL3* to inhibit flowering under stress conditions.

After 6 h of treatment, miR171c-5p levels were reduced and its cognate novel predicted target *APS reductase 3* was upregulated (Supplementary Table 8). *APS reductase 3* controls the rate-limiting step in sulfur assimilation. This enzyme was reported to be produced in high amounts after 4 h of light treatment in *A. thaliana* and when supplemented with 0.5% sucrose, its amount increased sevenfold (Kopriva et al., 1999). It is also known that continuous light treatments lead to increased sugar levels which act as mediators of light (Haque et al., 2015; Chen et al., 2019) and could enhance the *APS reductase* expression. These studies point toward the importance of *APS reductase* expression in response to light and sucrose treatments. Studies have shown that APS reductase is needed to synthesize additional cysteine required for glutathione biosynthesis. During oxidative stress in *A. thaliana*, the amount of oxidized glutathione increases and the reduced form of glutathione decreases which drives the expression of *APS reductase* (Leustek, 2002). Considering the impact of enhanced oxidative stress during high light stress, our data indicates an important role of miR171c-5p in the control of *APS3 reductase* transcript levels in order to promote the biosynthesis of reduced glutathione.

At the same time point, the upregulation of miR163 led to downregulation of one of its predicted target transcripts coding for a S-adenosyl-L-methionine-dependent methyltransferases superfamily protein (AT1G15125) (Supplementary Table 8). It is known that SAM dependent carboxyl methyltransferases are a family of plant enzymes that act on a variety of substrates such as salicylic acid, jasmonic acid and 7-methylxanthine to produce their methyl compounds (Ross et al., 1999). In conditions of high light stress, it is likely that the plant maintains its levels of SA and JA by reducing the levels of methyltransferases that could be necessary for defense and development (Kazan and Manners, 2011; Svyatyna and Riemann, 2012; Khan et al., 2015). After 6 h and 2 days of treatment, miR395a was downregulated accompanied by elevated levels of one of its novel putative targets encoding cellulose synthase like G3 (Supplementary Table 8). A previous study revealed differential regulation of this transcript in *cry1* mutants subjected to blue light (Folta et al., 2003) and in *phyB* mutants exposed to continuous monochromatic red light (Tepperman et al., 2004). Light receptors such as *cry1* and *phyB* perceive light and mediate growth control with the help of cellulose synthase which is involved in maintaining the strength and composition of cell walls (Bischoff et al., 2011; Le Gall et al., 2015). The upregulation of this transcript (6 h and 2 days) in response to high light treatment may lead to increased mechanical strength to withstand elevated turgor pressure. This hypothesis is supported by mutants that are defective in cellulose synthase like genes displaying an enhanced sensitivity to salt stress (Wang et al., 2016a; Zhang et al., 2016).

We further investigated sRNAs derived from lncRNA, *cis*- and *trans*-NATs, *TAS* and *PHAS* RNAs. We found 11 non-redundant, non-overlapping lncRNAs which produced DE sRNAs during the course of high light treatment. The transcripts of all parent lncRNA transcripts were undetectable in the sequencing data. We found two lncRNAs that differentially increased the production of sRNAs and the remaining 9 lncRNAs led to decreased sRNA production in high light samples compared to their respective controls (Supplementary Table 15). A study in rice may explain these observations where *Psi-LDMAR* siRNAs generated from the lncRNA *Long day specific male fertility associated RNA* (*LDMAR*) were able to downregulate the *LDMAR* transcript through RNA-dependent DNA methylation (RdDM) (Ding et al., 2012). We can speculate from this example that the steady state levels of siRNA producing lncRNA parent transcripts were maintained by their cognate siRNAs.

We also found 90 non-redundant *cis*-NATs pairs and 104 *trans*-NATs pairs that led to the production of differentially expressed nat-siRNA over the time course of high light treatment (Supplementary Table 16). After 6 h of treatment, we observed *cis*-nat-siRNAs being produced from two pairing transcripts. The transcript encoding *NUCLEOLIN LIKE 1* was upregulated and *glycosyl hydrolase 9C1* transcript levels remained unchanged. Salt stress causes elevated *NUCLEOLIN LIKE 1* transcript levels pointing to its role in rRNA processing during salt stress adaptation (Huang et al., 2018). Similar results were obtained in our data indicating an involvement of this gene in response to high light treatment. Another *cis*-NATs pair reduced the production of nat-siRNAs. While the upregulated transcript encodes for ARABIDOPSIS HOMOLOGUE OF YEAST BRX1-1 (AT3G15460) that plays a role in the maturation of the large ribosomal subunit and facilitates pre-rRNA processing, its pairing transcript encoding aluminum induced protein with YGL and LRDR motifs (AT3G15450) was reduced. Studies have shown that AT3G15450 is an auxin responsive transcript that was found to be downregulated by drought stress (Huang et al., 2008). Its downregulation is also observed in high light treatment, but the significance of its repression in stress remains unknown. After 2 days of high light treatment, NATs AT3G51238 and AT5G01595 paired with a transcript encoding flavanone 3-hydroxylase (AT3G51240) and FERRETIN 1 (AT5G01600), respectively. Both pairing transcripts as well as the derived nat-siRNAs were upregulated. It can be speculated that the upregulation of these transcripts is necessary for high light acclimation since flavanone 3-hydroxylase is known to promote flavonoid accumulation in high light (Pelletier and Shirley, 1996) and FERRETIN 1 is known to increase the photosynthetic performance of plants in response to oxidative stress (Briat et al., 2010). The upregulated levels of nat-siRNAs could be responsible for maintaining steady state levels of the parent transcripts expressed in response to high light treatment. This may occur by maintaining an equilibrium between the rate of transcription of the parent transcripts and the rate of subsequent nat-siRNAs generation. The *trans*-nat-siRNAs were mostly produced from pc:nc transcript pairs with pre-tRNAs being the most prominent nc pairing partner (Supplementary Table 17). Studies have shown how sRNAs derived from tRNAs and mRNAs can regulate post-transcriptional gene expression (De Lay and Garsin, 2016). At all the three time points, we found nat-siRNAs produced from single stranded regions of partially overlapping transcripts as well as from the double stranded regions of completely overlapping transcripts. The second most abundant nc pairing partner that led to differentially expressed *trans*-nat-siRNA were TE derived RNAs. TEs have the potential to mobilize and induce mutations in the host genome. Thus, plants have evolved special mechanisms to control the expression of TEs which are based on RNA silencing and chromatin modifications (Slotkin and Martienssen, 2007). Studies have confirmed that tRNA derived sRNAs can target endogenous TE (Martinez et al., 2017) and target other non-TE targets (Creasey et al., 2014; Cho, 2018).

We also detected differentially expressed genes and sRNAs that might regulate anthocyanin biosynthesis under high light conditions (Figure 5). For example, miR828 seems to be involved in two pathways regulating *PAP1* transcripts. In the indirect pathway, that was elucidated in sugar treated plants, miR828 triggers the production of *TAS4* derived ta-siRNAs which target and negatively control *MYB* transcription factors including *PAP1* and *PAP2* resulting in reduced anthocyanin production (Luo et al., 2012). PAP1 also has the ability to bind to the promoter region of the *TAS4* gene to induce its expression (Luo et al., 2012) indicating the existence of a negative autoregulatory loop between the PAP1-*TAS4* and ta-siRNAs in response to high light. In the direct pathway, miR828 is able to directly target the *PAP1* transcript and its overexpression causes reduced *PAP1* transcript levels and represses anthocyanin biogenesis (Yang et al., 2013). However, an additional pathway involving HY5 induced transcriptional activation of *PAP1* was observed (Shin et al., 2013). We detected upregulation of *HY5* encoding a transcriptional regulator that enhances *PAP1* transcription by binding to G- and ACE-boxes in the *PAP1* promoter. This regulation might explain increased steady state levels of *PAP1* transcripts in our study. In support of this positive regulation of *PAP1* by HY5 and an anticipated increase in anthocyanin production, we observed increasing transcript levels of downstream genes encoding enzymes such as DFR, CHS and ANS that act in the anthocyanin biosynthetic pathway. Apart from its role in the sugar and high light response, miR828 is also known to trigger the production of *TAS4* derived *ta-siRNAs* in response to Pi deficiency (Hsieh et al., 2009). We found more than twofold upregulation of members acting in the regulation of anthocyanin production: miR828, *TAS4* transcript, *TAS4*-derived ta-siRNAs, *PAP1*, and *PAP2*, *HY5*, *DFR*, *CHS* and *ANS*. Taken together, our results support previously reported studies (Hsieh et al., 2009; Luo et al., 2012; Yang et al., 2013) and explains the existence of a negative autoregulatory loop. The model expands the current knowledge on regulatory components of the anthocyanin biosynthetic pathway by the identification of *HY5* and *PAP1* that may lead to elevated anthocyanin levels in response to high light.

Consequently, the proposed model for anthocyanin biosynthesis as well as the high number of identified miRNAs, sRNAs derived from *cis*-and *trans*-NAT gene pairs and from lncRNAs provide a fundamental base to elucidate sRNA-controlled gene regulatory networks underlying molecular adaptations of high light induced acclimation responses.

## Data Availability Statement

The original contributions presented in the study are publicly available. This data can be found here: The raw Illumina sRNA and mRNA sequencing data is deposited in NCBI SRA database with the ID PRJNA653584.

## Author Contributions

WF designed the research. BT performed the research with the help of MA and KH. BT, MA, KH, OT, and WF analyzed the data. BT, OT, and WF wrote the manuscript. All authors read and approved the final manuscript.

## Conflict of Interest

The authors declare that the research was conducted in the absence of any commercial or financial relationships that could be construed as a potential conflict of interest.

## Footnotes

1 https://usegalaxy.org/

2 https://www.arabidopsis.org

3 https://araport.org/

4 https://CRAN.R-project.org/package=ggplot2

5 ftp://ftp.arabidopsis.org/home/tair/Genes/SmallRNAsCarrington/

## Supplementary Material

Supplementary Table 1 List of stem loop qPCR oligonucleotides used in this study.

Supplementary Table 2 Mapped mRNA sequencing reads after adapter trimming in control and high light treated samples (biological triplicates).

Supplementary Table 3 Mapped sRNA sequencing reads from specific sRNA producing RNA classes in control and high light treated samples.

Supplementary Table 4 The size distribution of sRNAs (reads per million) from control and high light treated samples after adapter trimming.

Supplementary Table 5 DE miRNAs upon 3 h, 6 h, and 2 days of high light acclimation, respectively. Evolutionarily conserved miRNA families are highlighted in orange.

Supplementary Table 6 Normalized read counts and log2FC of all miRNAs upon 3 h, 6 h, and 2 days of high light acclimation, respectively.

Supplementary Table 7 High light-responsive DE miRNAs in *Arabidopsis*. MiRNAs were classified as DE miRNAs when log2FC ≥ 1 and ≤−1 (Benjamini-Hochberg corrected *p*-value ≤ 0.05).

Supplementary Table 8 Targets of all DE miRNAs at the three time points predicted using psRNATarget Analysis Server. N/A = No significant fold change.

Supplementary Table 9 NcRNA targets of all DE miRNAs at three time points predicted using psRNATarget Analysis Server. N/A = No significant fold change.

Supplementary Table 10 List of targets of DE miRNAs found to be consistently present throughout the course of high light treatment and a venn diagram depicting the overlap of all the targets.

Supplementary Table 11 All mRNAs and their normalized read counts (triplicates) acquired from mRNA sequencing data of control and high light treated samples at 3 h, 6 h, and 2 days.

Supplementary Table 12 Detailed list of all DE mRNAs acquired from mRNA sequencing data (control and 3 h, 6 h and 2 days of high light treated samples.

Supplementary Table 13 Table representing previously known validated or predicted miRNA targets.

Supplementary Table 14 Gene Ontology term enrichment analysis for putative targets of DE miRNAs after 3 h, 6 h, and 2 days of high light acclimation.

Supplementary Table 15 DE sRNAs derived from non-overlapping lncRNAs. The sRNA and lncRNA sequencing data at different time points are shown in the sub-tables.

Supplementary Table 16 DE sRNAs generated from *cis*-NAT pairs. The sRNA and *cis*-NAT sequencing data at different time points are shown in the sub-tables.

Supplementary Table 17 DE sRNAs generated from *trans*-NAT pairs. The sRNA and *trans*-NAT sequencing data at different time points are shown in the sub-tables.

Supplementary Table 18 DE sRNAs generated from *TAS* transcripts. The sRNA and *TAS* sequencing data at different time points are shown in the sub-tables.
